# A Glimpse into the World of Integrative and Mobilizable Elements in Streptococci Reveals an Unexpected Diversity and Novel Families of Mobilization Proteins

**DOI:** 10.3389/fmicb.2017.00443

**Published:** 2017-03-20

**Authors:** Charles Coluzzi, Gérard Guédon, Marie-Dominique Devignes, Chloé Ambroset, Valentin Loux, Thomas Lacroix, Sophie Payot, Nathalie Leblond-Bourget

**Affiliations:** ^1^UMR1128 DynAMic, Institut National de la Recherche Agronomique, Université de Lorraine,Vandœuvre-lès-Nancy, France; ^2^UMR7503 Laboratoire Lorrain de Recherche en Informatique et ses Applications, Centre National de la Recherche Scientifique, Université de Lorraine,Vandœuvre-lès-Nancy, France; ^3^UR1404 Mathématiques et Informatique Appliquées du Génome à l’Environnement, Institut National de la Recherche Agronomique, Université Paris-Saclay,Jouy-en-Josas, France

**Keywords:** mobilizable elements, relaxase, TcpA coupling protein, conjugation, *Streptococcus*

## Abstract

Recent analyses of bacterial genomes have shown that integrated elements that transfer by conjugation play an essential role in horizontal gene transfer. Among these elements, the integrative and mobilizable elements (IMEs) are known to encode their own excision and integration machinery, and to carry all the sequences or genes necessary to hijack the mating pore of a conjugative element for their own transfer. However, knowledge of their prevalence and diversity is still severely lacking. In this work, an extensive analysis of 124 genomes from 27 species of *Streptococcus* reveals 144 IMEs. These IMEs encode either tyrosine or serine integrases. The identification of IME boundaries shows that 141 are specifically integrated in 17 target sites. The IME-encoded relaxases belong to nine superfamilies, among which four are previously unknown in any mobilizable or conjugative element. A total of 118 IMEs are found to encode a non-canonical relaxase related to rolling circle replication initiators (belonging to the four novel families or to MobT). Surprisingly, among these, 83 encode a TcpA protein (i.e., a non-canonical coupling protein (CP) that is more closely related to FtsK than VirD4) that was not previously known to be encoded by mobilizable elements. Phylogenetic analyses reveal not only many integration/excision module replacements but also losses, acquisitions or replacements of TcpA genes between IMEs. This glimpse into the still poorly known world of IMEs reveals that mobilizable elements have a very high prevalence. Their diversity is even greater than expected, with most encoding a CP and/or a non-canonical relaxase.

## Introduction

Conjugative elements drive horizontal gene transfer between bacteria, and therefore play a key role in bacterial evolution. These mobile elements encode all factors needed for their autonomous transfer by conjugation. The conjugative transfer of various plasmids from Gram-negative (G-) bacteria, especially proteobacteria, is well understood (Cabezon et al., 2015; Chandran Darbari and Waksman, 2015; Ilangovan et al., 2015) and proceeds as follows. The plasmid DNA is recognized and processed by the relaxosome, a complex that includes a relaxase protein encoded by the element. Up to now, six superfamilies of relaxases (MobC, MobF, MobH, MobP, MobQ, and MobV) are known to be encoded by conjugative plasmids from proteobacteria (Garcillan-Barcia et al., 2009) and are referred in this paper as canonical relaxases. The relaxase catalyzes a site- and strand-specific cleavage of the origin of transfer (*oriT*) at the *nic* site of its cognate plasmid. The relaxase-tethered DNA is then recruited to the coupling protein (CP) belonging to the VirD4 family. The CP interacts with a multi-protein complex known as a type IV secretion system (T4SS) which spans the cellular envelope of the donor cell. The CP and T4SS subsequently translocate the single-strand DNA-relaxase complex through membranes and cell walls into the recipient cell. The nicking of *oriT* by the relaxase also initiates a rolling-circle replication (RCR) of the plasmid by cellular enzymes so that the donor cell retains the plasmid and the recipient cell acquires the plasmid. In addition to conjugative plasmids, other autonomous elements called integrative and conjugative elements (or ICEs) are found to be integrated in the chromosomes of bacteria. ICEs encode their own excision, transfer by conjugation, and integration (for reviews see Burrus et al., 2002; Bellanger et al., 2014). Apart from the excision and integration steps that are catalyzed by a tyrosine recombinase, a serine recombinase or a DDE transposase, the conjugative transfer of most ICEs is assumed to resemble that of plasmids of G- bacteria, and therefore to involve a relaxase, a CP and a T4SS machinery.

Many other mobile elements, known as mobilizable elements, hijack the conjugative machinery of unrelated conjugative elements (Francia et al., 2004; Garcillan-Barcia et al., 2009; Meyer, 2009). The best known mobilizable elements are the mobilizable plasmids from G- bacteria. While a very few mobilizable plasmids encode a CP, they never encode any other protein belonging to the T4SS. Almost all of them encode a relaxase from one of the six canonical superfamilies, but which is distantly related to those of the conjugative plasmids. These relaxases recognize and cut their cognate *oriT* (Francia et al., 2004; Meyer, 2009). They then recruit the CP and/or T4SS of a conjugative element to mobilize *in trans* the non-autonomous element. In addition to mobilizable plasmids, some integrative elements known as integrative and mobilizable elements (IMEs) also transfer by mobilization. IMEs encode their own excision and integration but carry only some of the sequences or genes necessary for their conjugative transfer (for a review, see Bellanger et al., 2014). Most of the very few IMEs described so far carry their own *oriT* and encode their own relaxase, but none encode a CP or any protein belonging to the T4SS. Other previously described IMEs carry their own *oriT* but do not encode any protein involved in conjugation. To date, very few genomes searches have focused explicitly on IMEs so their prevalence and diversity are essentially unknown (Brochet et al., 2008; Bellanger et al., 2014). However, Guglielmini et al. (2011) performed an extensive search for relaxases and conjugation modules in 1124 archaeal and bacterial genomes, and identified many isolated relaxase genes on chromosomes, which suggests that IMEs are the most prevalent elements that transfer by conjugation.

While conjugation and mobilization mechanisms are well known in G- proteobacteria, they are poorly documented in all other bacterial clades, including the Firmicutes, a major group of Gram-positive (G+) bacteria. The conjugative plasmids and ICEs from firmicutes encode T4SSs belonging to two families, FA and FATA, that have not been found in G- bacteria (Guglielmini et al., 2014). The FATA T4SS was found to be associated with classical CPs (VirB4) and with relaxases belonging to the canonical MobP, MobQ, and MobC superfamilies (Guglielmini et al., 2013; Ambroset et al., 2016). In contrast, most plasmids and ICEs with FA T4SSs encode TcpA CPs instead of VirD4. The TcpA CPs are related to FtsK, the double strand DNA translocase involved in DNA segregation during cell division (Guglielmini et al., 2013; Ambroset et al., 2016). Furthermore, all the plasmids and ICEs that encode FA T4SSs and TcpAs CPs encode non-canonical relaxases. Thus, the pCW3 conjugative plasmid from the Firmicute *Clostridium perfringens*, which encodes a FA T4SS and a TcpA CP, was recently shown to encode a novel type of relaxase related to tyrosine recombinase (Wisniewski et al., 2016). In the same way, all ICEs, which encode a FA T4SS and a TcpA CP, encode a relaxase belonging to the non-canonical MobT superfamily. The MobT relaxases are related to a family of RCR initiators involved in the intracellular replication and maintenance of small plasmids (Guglielmini et al., 2013; Ambroset et al., 2016). MobT encoded by ICE*Bs1* is involved in both conjugative transfer and in replication of the excised ICE [for a review, see (Auchtung et al., 2016)]. It should be mentioned that most families of relaxases involved in the RCR of small plasmids or viruses are clearly distinct from and perhaps unrelated to the superfamilies of relaxases found in conjugative and mobilizable elements. Besides the MobT relaxases encoded by the ICEs with FA T4SS and TcpA CP, the only other exception corresponds to PF01446 RCR initiators of some mobilizable plasmids of firmicutes that are involved in both plasmid replication and mobilization by ICEs encoding TcpA CPs (Naglich and Andrews, 1988; Showsh and Andrews, 1999; Lee et al., 2012).

Streptococci are G+ bacteria belonging to the Firmicutes. Genome and phylogenetic analyses have shown that a large proportion of the streptococcal genomes has experienced horizontal gene transfer (Richards et al., 2014). Previously, our extensive analyses of the genomes of 124 strains belonging to 27 streptococcal species revealed a high prevalence of ICEs (Ambroset et al., 2016), suggesting that a significant fraction of these transfers could be due to those elements. Furthermore, a comprehensive search of IMEs performed on eight available genomes of the firmicute *Streptococcus agalactiae* revealed twelve IMEs (Brochet et al., 2008). Surprisingly, this search also detected nine genomic islands which possess a complete integration/excision module and encode a putative CP belonging to TcpA family, but which do not encode any proteins related to known relaxases. Therefore, these elements could correspond to IMEs encoding a relaxase that is very distantly related or unrelated to known relaxases. A preliminary reanalysis of these nine elements from *S. agalactiae* revealed that they encode proteins related to RCR initiators from plasmids or viruses, suggesting that these elements are probably IMEs encoding novel types of relaxases related to RCR initiators. In this study, we searched for IMEs in the 124 publicly available complete genomes of *Streptococcus* that were previously used for the ICE search. IMEs were defined by the combined presence of putative integrases and relaxases (related to classical relaxases or to RCR initiators), the eventual presence of putative CPs (VirD4 or TcpA), and the absence of T4SSs. CDSs encoding these signature proteins were localized on the chromosomes and their boundaries and integration site of IMEs were identified. This study (i) gives a general overview of the very high prevalence and diversity of putative IMEs within streptococci, (ii) identifies their numerous specific sites of insertion, (iii) reveals that most IMEs harbor a versatile and compact mobilization module that encodes a non-canonical relaxase related to RCR initiators and generally a non-canonical CP related to FtsK.

## Materials and Methods

### Genomes Examined and Database of Reference Proteins

The dataset of the 124 complete chromosomes from *Streptococcus* species available at the start of the present study was taken from Genbank^1^. This initial database of reference proteins contains signature proteins from ICEs and the few IMEs reported for Firmicutes in the literature. It includes protein sequences from 50 tyrosine integrases, 13 serine integrases, 2 DDE transposases, 50 relaxases, 37 CPs, and 26 VirB4 proteins (a T4SS ATPase). This last protein has never been found in any mobilizable elements and is used here as the main criterion for differentiating between ICEs and IMEs at the detection step.

### Workflow

The overall workflow of our search strategy to detect and characterize IMEs in streptococcal chromosomes was described previously (Ambroset et al., 2016). This workflow allows (i) detecting ICEs from the presence of signature CDSs grouped on the genome, (ii) identifying ICE insertion sites and (iii) delineating ICEs. The workflow was adapted to IME detection by modifying the signature CDSs in step (i): a putative IME is detected when no VirB4 CDS is present and when an integrase CDS is found in the vicinity of a relaxase CDS. Steps (ii) and (iii) were conducted in the same way as for ICEs.

When an IME signature CDS was missing or incomplete (pseudogene), the corresponding complete CDS encoded by the closest known IME was taken and compared to the putative defective one by tBlastN in order to detect possible genome annotation errors (e.g., identification of an authentic gene as a pseudogene most frequently due to the presence of a type II intron within the gene or mis-identification of the “start” codon). Because before this work, the known IMEs did not encode a CP, those elements carrying an integrase gene, a relaxase gene, and a CP pseudogene were considered as IMEs. Moreover, our workflow detects elements containing only an integrase and a CP CDS but apparently no relaxase gene or pseudogene. In such cases, an exhaustive manual analysis was performed to search for new relaxase genes, in particular for genes encoding proteins related to RCR initiators. Newly found relaxases were added to the database of reference proteins and step (i) was reiterated on all genomes. In summary, we considered as IMEs all elements delimited by direct repeats and containing at least one CDS for one complete integrase as well as one complete relaxase, but no CDS for VirB4 or other proteins of the T4SS.

### Denomination of IMEs

Each IME name indicates by letters and numbers the species and strain of the host bacteria. When an IME encodes a site-specific integrase, its denomination also specifies the name of the target gene. IMEs marked with an asterisk are not integrated in their primary site but in a secondary one as previously observed for ICEs (Ambroset et al., 2016).

### Domain Composition Analysis and Tree Construction

The retrieval of the domain composition of all IME signature proteins from Uniprot annotations was done in batch using the BioMart Central Portal^2^. *De novo* conserved domain search (CD-search^3^) and/or PSI-Blast analyses were performed when no data was available through BioMart. The correspondence with Mob families was established using the CONJscan-T4SSscan program^4^ (Guglielmini et al., 2011). This tool is no longer accessible but should soon be available on the Pasteur Galaxy server.

The signature proteins were aligned using Clustal omega with default parameters (Sievers et al., 2011). The trees of signature proteins were built with MEGA (Tamura et al., 2013) using (i) maximum likelihood (ML) based on the JTT (Jones–Taylor–Thornton) model including amino acid empirical frequencies (partial deletion of gaps and missing data at 80% cutoff, Gamma distribution in five categories, allowance for invariant sites), and (ii) BioNJ methods with the Poisson model (Gouy et al., 2010). The branch support of the groupings was estimated using bootstrap (100 replicates).

### Protein Clustering and Signature Proteins Associations

Protein clustering at 90 and 40% sequence identity was performed using BLASTclust^5^ (Alva et al., 2016) with default parameters. Circos^6^ was used to visualize the signature protein associations (Krzywinski et al., 2009). The functional annotation of IMEs was performed using Agmial as described previously (Ambroset et al., 2016).

## Results

### Prevalence of IMEs within Streptococcal Chromosomes

The prevalence of IMEs was studied in a large set of species (27 *Streptococcus* species) and strains (*n* = 124). This exhaustive examination led to the identification of 144 IMEs. Their sizes ranged from 5 to 18 kb (Supplementary Table S1) except for IME_*Sparas15912_rpsI* which was 53 kb. The larger size of IME_*Sparas15912_rpsI* has likely resulted from a tandem ICE-IME insertion in the element. Within streptococcal genomes, this study showed that IME sizes (mean = 10 kb) were generally smaller than those of ICEs (mean = 41 kb). This difference is reminiscent of that observed between mobilizable and conjugative plasmids (Smillie et al., 2010).

Ten IMEs were found to be integrated in tandem accretion with other IMEs (IME1-IME2) or with ICEs (ICE-IME or ICE-IME1-IME2) (Supplementary Table S1) and many with decayed elements (data not shown). Nine tandems were found to be integrated in the 3′ end of *rpsI*, *rpmG*, or *rplL*. The last one was integrated in a secondary site inside IME_*Sparas15912_rpsI.* All IMEs in accretion in the same site had a tyrosine integrase but some decayed IMEs in accretion were found to encode serine integrases (data not shown). These integrases had from 21 to 69% sequence identity, showing that there was no particular relatedness between integrases encoded by elements in accretion.

More than half (*n* = 78) of the streptococcal chromosomes contained at least one IME. As seen in Supplementary Table S1, the occurrence of IMEs varied within species and strains. For instance, most of the 20 *S. pyogenes* chromosomes were deprived of IMEs, whereas the 3 *S. anginosus* chromosomes each contained from 4 to 6 IMEs. Interestingly, the genomes that contained the lowest numbers of IMEs (*S. pyogenes* with an average of 0.1 IME per genome) also contain few ICEs (0.1 ICE per genome). The opposite was also true; the genomes from *S. anginosus* were among those containing the highest numbers of IMEs (mean = 4.7) as well as ICEs (mean = 4.3).

### Diversity of Integration Modules and Integration Sites of Streptococcal IMEs

Almost all of the integrase genes of streptococcal IMEs were located at one end of the element and were outward facing. These integrases belonged to two unrelated superfamilies: tyrosine integrases and serine integrases.

#### Diversity of IME Tyrosine Integrases and of Their Integration Sites

**FIGURE 1:**
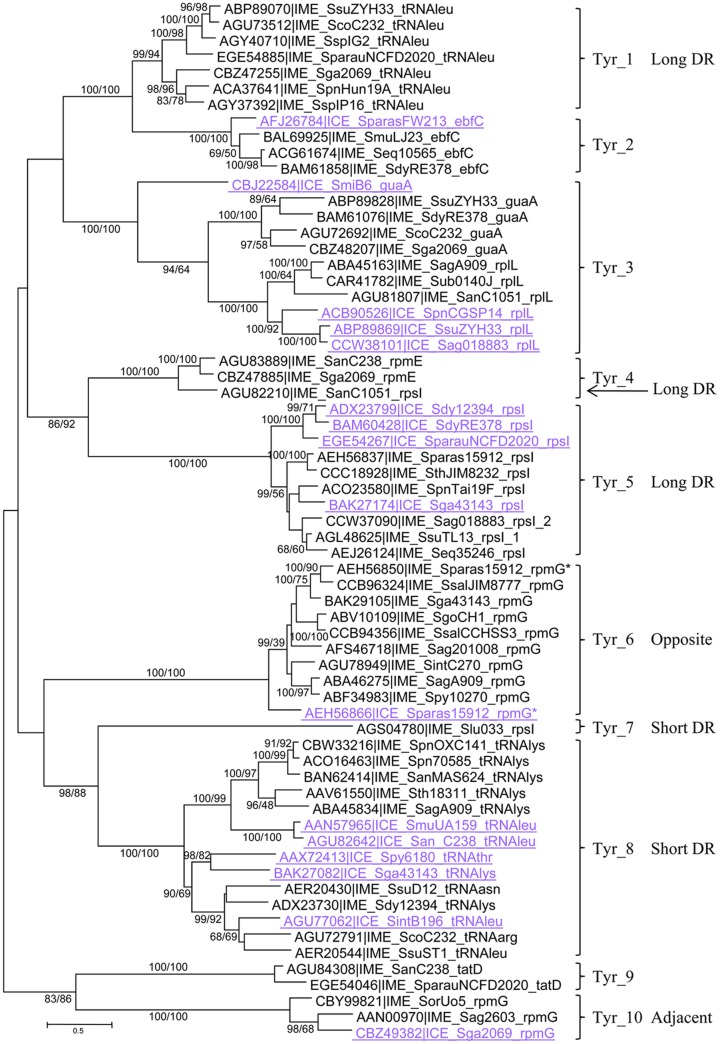
**Phylogenetic tree of tyrosine integrases.** One representative of each 90% protein identity cluster from integrative and mobilizable elements (IMEs) (in black) and one representative of each 90% protein identity cluster of tyrosine integrases from ICEs targeting the same site as IMEs (in mauve and underlined) are presented in the ML tree. Bootstrap values (BioNJ/ML) are given only when they exceed 50 for both analyses. The target gene is mentioned in the IME/ICE names. Tyrosine integrases sharing more than 40% sequence identity and therefore belonging to the same family are merged with brackets. These families are distinguished with different numbers. The DR length or integrase position is indicated to distinguish tyrosine integrases belonging to different families but targeting the same genes. Refer to Supplementary Table S1 for IME and strain details.

**FIGURE 2:**
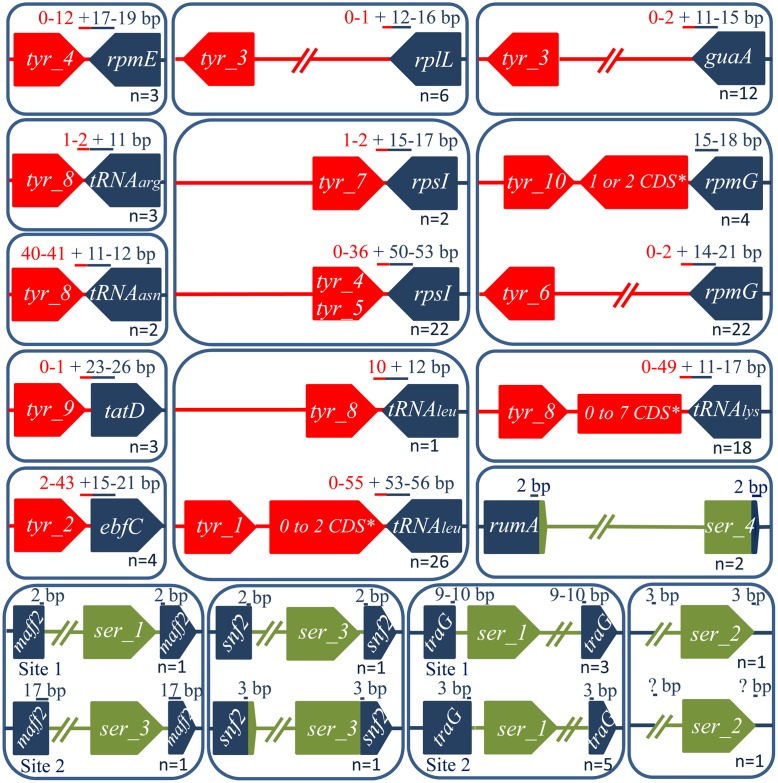
**Integrative and mobilizable element integration loci and their position relative to the integrase CDSs.** Tyrosine and serine integrase genes are shown in red and green, respectively. The target genes (dark blue) encode, respectively: *ebfC* [nucleoid associated protein], *guaA* [GMP synthase], *maff2* [conserved membrane protein of ICEs belonging to Tn*5252* superfamily], *rpsI* [S9 ribosomal protein], *rplL* [L7/L12 ribosomal protein]*, rpmE* [L31 ribosomal protein], *rpmG* [L33 ribosomal protein]*, rumA* [23S rRNA (uracil-5-) methyltransferase], *snf2* [helicase of ICEs belonging to Tn*5252* superfamily], *tatD* [DNAse], *traG* [VirD4 CP gene from ICEs belonging to Tn*5252* superfamily]. The DR size (in bp) within the target gene is indicated in blue and the one outside is in red. The number of ICEs integrated in a given site is marked at the bottom of each box.

Tyrosine integrases were detected in more than 89% of the IMEs (*n* = 128/144). Both phylogenetic analysis and clustering of the tyrosine integrases at 40% sequence identity allowed them to be classified in 10 distinct families (**Figure 1**). In most cases, tyrosine integrases belonging to the same family catalyze integration in the same gene (**Figures 1**, **2**). For instance, all tyrosine integrases allocated to family Tyr_1 target the 3′ end of the tRNAleu gene. Three exceptions to this rule were observed. First, the Tyr_3 family includes tyrosine integrases targeting two distinct insertion genes (*guaA* or *rplL*). Phylogenetic analysis of these integrases suggests an evolution from *guaA* specificity to *rplL* specificity (**Figure 1**). Second, the Tyr_4 family gathers all integrases targeting *rpmE* and one integrase targeting *rpsI*. Phylogenetic analysis of these integrases and the sister Tyr_5 group of integrases suggests an evolution from *rpsI* specificity to *rpmE* specificity. Third, integrases of family Tyr_8 catalyze integration within genes encoding four different tRNAs (tRNAasn, tRNAleu, tRNAlys, and tRNAarg). Although catalyzing integration in distinct tRNA genes, all integrases from family Tyr_8 share the ability to generate short direct repeats (DRs).

In the majority of cases, each insertion gene is specifically targeted by closely related tyrosine integrases grouped in one unique family (**Figures 1**, **2**). Exceptions were *rpsI, rpmG*, and the tRNAleu genes that are targeted by integrases belonging to several families. More specifically, *rpsI* is targeted by tyrosine integrases belonging to three distinct families; the integrases from families Tyr_4 and Tyr_5 lead to the formation of long DRs whereas those from family Tyr_7 all have short DRs (**Figure 2**). Similarly, the integrases targeting the tRNAleu gene belong to two distinct families; the integrases from family Tyr_1 have long DRs, and those from family Tyr_8 have short DRs. Finally, two families of integrases catalyze integration in *rpmG* and lead to two distinct architectures after integration: genes encoding integrases from family Tyr_10 are adjacent to *rpmG*, whereas those encoding integrases from family Tyr_6 are at the opposite end of the IME.

In summary, the analysis of the integration loci of 128 IMEs harboring a tyrosine integrase shows that these integrases specifically target 11 distinct genes, mainly (109/128) at the 3′ end of genes encoding tRNAs or ribosomal proteins. More rarely (19/128), tyrosine integrases from IMEs catalyze integration at the 3′ or 5′ end of other protein-encoding genes (*guaA*, *tatD*, or *ebfC*). This study has therefore extended the known list of possible integration sites for streptococcal IMEs with tyrosine integrases: only four integration sites were previously identified (*oriT* from ICEs belonging to Tn*916* and ICE*St3* families, 3′ *rpsI*, 3′ tRNALys gene and 3′ *rpmG*) (Brochet et al., 2008; Puymege et al., 2015; Lorenzo-Diaz et al., 2016).

#### Diversity of IME Serine Integrases and of Their Integration Sites

Our collection of IMEs contains 16 IMEs encoding a serine integrase. For two IMEs (IME_*Sol3089_ND* and IME_*SsalCCHSS3_ND*) encoding related integrases belonging to family Ser_2, we were unable to determine the specificity of integration because these two IMEs were integrated in two distinct intergenic regions (**Figure 2**). Two other IMEs with serine integrases were found to be integrated within the bacterial gene *rumA*, which is already known to be a common integration site for ICEs with serine integrases (Ambroset et al., 2016). All other 12 IMEs were found to be integrated within conserved genes from ICEs belonging to the Tn*5252* superfamily: *traG* (encoding a VirD4 CP), *maff2* (encoding a membrane protein), and *snf2* (encoding a helicase) (Supplementary Table S1), thereby leading to gene disruption.

Both phylogenetic analysis and 40% sequence identity clustering of the serine integrases indicate that they may be classified in four distinct families (Ser_1 to 4, **Supplementary Figure S1**). A large majority of the serine integrases sharing the same integration specificity were grouped in the same family. For example, the integrases targeting the *traG*, *snf2*, and *rumA* genes were grouped in families Ser_1, Ser_3, and Ser_4, respectively. Exceptions were the two serine integrases targeting *maff2* that were found in two distinct families: the one belonging to family Ser_1 shows a close relatedness with integrases targeting *traG*, whereas the one belonging to family Ser_3 is related to integrases targeting *snf2*. These two integrases catalyze integration in two different locations within *maff2* and lead to the generation of distinct DRs (**Figure 2**). This variability of integration within the same gene was also observed for integration inside *traG*. The two IMEs specific to *snf2* are integrated in the same location within *snf2*, but the recombination site of the IME is located within the integrase gene for IME_*Spy2096_SNF2*, whereas it is adjacent to the 3′ end of the integrase gene for IME_*SsuT15_SNF2* (**Figure 2**).

#### Comparison of the Recombination Modules of IMEs and ICEs

Our collection of IMEs (this work) and ICEs (Ambroset et al., 2016) from streptococci allowed us to compare the recombination modules of these two types of element. Both of them encode serine and tyrosine integrases, and their diversity is similar in IMEs and in ICEs. Indeed, we detected 18 distinct specificities for integrases from streptococcal IMEs (17 targeting a specific site and one with unknown specificity) and at least 17 for the ICEs. However, in contrast to the ICEs, no DDE transposase was found in any of the IMEs.

As shown in **Figure 1** and **Supplementary Figure S1**, several integrase families (such as Tyr_2, Tyr_3, Tyr_5, Tyr_6, Tyr_8, Tyr_10, and Ser_4) grouped both integrases from IMEs and ICEs. For three specificities: *ebfC* (Tyr_2), *rplL* (Tyr_3), and *rpmG* opposite (Tyr_6), tyrosine integrases from IMEs and ICEs belong to closely related sister groups. In family Tyr_5, the tyrosine integrases from IMEs targeting *rpsI* are mixed with those of the ICEs, and in cluster Ser_4 the serine integrase targeting *rumA* from IME*_SpnAP200_rumA* shares 91–93% identity with those of two ICEs. Altogether, these results suggested that exchange of integration modules between ICEs and IMEs are frequent.

### New Relaxase Families Related to RCR Initiator Proteins in IMEs

A total of 154 relaxase genes were detected within IMEs (including three pseudogenes in IMEs harboring two relaxases). Based on their domain composition, the relaxases were classified in nine distinct superfamilies (**Table 1**). Among the four most prevalent relaxase superfamilies, only the Rel_PF02486 superfamily was recognized by the CONJscan-T4SSscan analysis as belonging to a known type of relaxase (MobT). The three others were novel superfamilies of relaxases characterized by the following domains: PF01719 (Rel_PF01719), PHA00330 (Rel_PHA00330) and by the combination of PF001719 and PF00910 (Rel_PF001719-PF00910). The Rel_PF02407 superfamily was the fourth novel superfamily identified in this study. All these new relaxase superfamilies discovered in IMEs harbored domains (PF01719, PHA00330, or PF02407) that were previously found exclusively in RCR initiators from viruses or plasmids (Ebisu et al., 1995; Bachrach et al., 2004; Gibbs et al., 2006; Lorenzo-Diaz et al., 2014). They were assumed to correspond to novel relaxase superfamilies since all these proteins were associated with an integrase and a large majority of these non-canonical relaxases (or all for PF02407) were associated with a CP in our IME collection. Four other relaxase superfamilies described in **Table 1** (Rel_PF03389, Rel_PF01076, Rel_PF13814, and Rel_PF03432) were recognized by the CONJscan-T4SSscan server as the MobQ, MobV, MobC, and MobP superfamilies, respectively.

**Table 1:** Relaxase superfamilies based on domain composition.

Superfamily name	Domain(s) ID^∗^	Domain name(s)	Conjscan domain	Number found	Number of clusters
Rel_PF02486/MobT	PF02486	Rep_trans	MobT	55	6
Rel_PF01719	PF01719	Rep_2	No hit	35	1
Rel_PH00330	PHA00330	Not applicable	No hit	21	3
Rel_PF01719-PF00910	PF01719 + PF00910	Rep_2 +RNA_helicase	No hit	15	4
Rel_PF03389/MobQ	PF03389	MobA_MobL	MobQ	12	1
Rel_PF01076/MobV	PF01076	Mob_Pre	MobV	11	1
Rel_PF02407	PF02407	Viral-Rep	No hit	2	1
Rel_PF13814/MobC	PF13814	Replic_Relax	MobC	2	1
Rel_PF03432/MobP	PF03432	Relaxase	MobP	1	1

*^∗^All domains are described in the Pfam classification except for domain PHA00330 that was found in the NCBI Conserved Domain classification without any Pfam equivalent.*

On the basis of their relaxase content, the IMEs were grouped in nine classes (IME_Class_1 to 9) encoding a unique relaxase, one for each superfamily of relaxase (**Table 2**). An additional class (IME_Class_10) contained 10 IMEs that carry 2 relaxases: one belonging to the Rel_PF02486/MobT and the other to the Rel_PF01076/MobV. Each of the two relaxase superfamilies present in this class also exists as standalone relaxases in IME_Class_1 and IME_Class_6.

**Table 2:** Diversity of the relaxases and CPs associated with serine and tyrosine integrases.

Integrase type (number)	Relaxase superfamily (number)	CP superfamily (number)	IME class
Tyrosine integrase (128)	Rel_IME_1/MobT(45)	TcpA (24) or none (21)	Class_IME_1
	Rel_IME_2 (35)	TcpA (34^∗^) or none (1)	Class_IME_2
	Rel_IME_3 (21)	TcpA (19^∗^) or none (2)	Class_IME_3
	Rel_IME_4 (15)	TcpA (4) or none (11)	Class_IME_4
	Rel_IME_1/MobT (10^∗^) + Rel_IME_6/MobV(10^∗^)	None	Class_IME_10
	Rel_IME_7 (2)	TcpA (2)	Class_IME_7
Serine integrases (16)	Rel_IME_5/MobQ (12)	None	Class_IME_5
	Rel_IME_8/MobC (2)	VirD4 (2)	Class_IME_8
	Rel_IME_6/MobV (1)	None	Class_IME_6
	Rel_IME_9/MobP (1)	None	Class_IME_9

*^∗^Number includes pseudogenes.*

Within each superfamily of relaxases, the diversity was estimated by phylogenetic analyses and 40% sequence identity clustering. The most abundant superfamily, Rel_PF02486/MobT (**Supplementary Figure S2**), includes six families (Rel_PF02486_1 to 6), among which the family PF02486_3 was associated with a Rel_PF01076/MobV in IME_Class_10. In contrast, the 35 relaxases from the Rel_PF01719 superfamily are closely related and were therefore clustered in a unique family (**Supplementary Figure S3**). The same was true for the 12 relaxases of the Rel_PF03389/MobQ superfamily (data not shown). The 21 members of the Rel_PHA00330 superfamily were grouped into three families (**Supplementary Figure S4**), and the Rel_PF01719-PF00910 superfamily (15 members) analysis yielded four families (**Supplementary Figure S5**). Finally, the superfamily Rel_PF01076 was clustered in two families, with the Rel_PF01076_1 being always found in IME_Class_10. Overall, the IME relaxases showed a great diversity: they were classified in nine distinct superfamilies subdivided in 20 families (**Table 1**). It should be noted that the diversity of IME relaxases largely exceeds that of ICEs, since ICEs are classified in three superfamilies/eight families according to the same criteria (Ambroset et al., 2016). Only two superfamilies of relaxases corresponding to Rel_PF02486/MobT and Rel_PF03432/MobP are encoded by both IMEs and ICEs. However, our analysis showed that within these superfamilies, the relaxases of IMEs always belong to clearly distinct families from those including ICE relaxases. Moreover, whereas the number of Rel_PF02486/MobT relaxases found for ICEs and IMEs are similar, it can be stressed that Rel_PF03432/MobP relaxases constitute the most abundant superfamily in ICEs (62/105 relaxases) and the least abundant in IMEs (only 1/154 relaxases).

### Half of the Streptococcal IMEs Encode a CP

Prior to the present study, none of the previously known or predicted IMEs encode a CP (Bellanger et al., 2014), and the few CPs that are known to be encoded by mobilizable plasmids belong to the VirD4 superfamily (Garcillan-Barcia et al., 2009). Surprisingly, our results show that more than half of the streptococcal IMEs encode a CP (85/144 including 11 pseudogenes of CP) and that almost all these CPs do not belong to the canonical VirD4 family. Indeed, only two IMEs encode a VirD4 CP characterized by a C-terminal VirD4 domain (COG3505 in the NCBI CDD classification). All others (72 proteins excluding pseudogenes) were found to display a unique PF01580 “FtsK-SpoIIIE” catalytic domain and to be more closely related to FtsK (a DNA translocase involved in DNA segregation during cell division) than to the canonical VirD4. According to the CONJscan-T4SSscan analysis, these proteins belong to a particular superfamily of CP named TcpA, found only in Firmicutes (Guglielmini et al., 2013). Reconstruction of their phylogeny and 40% identity clustering allowed their classification in 12 distinct families designated TcpA_1 to TcpA_12 (**Supplementary Figure S6**). The three most abundant families, TcpA_4, TcpA_7, and TcpA_12, contained 11, 11, and 30 proteins, respectively. The nine others were found from only 1 to 6 IMEs.

The two superfamilies (VirD4 and TcpA) of CPs were found in both ICEs (Ambroset et al., 2016) and IMEs with different prevalence. Within *Streptococcus* genomes, VirD4 CPs are encoded by 61/105 ICEs and by only the 2 IMEs belonging to IME_Class_8. In contrast, TcpA CPs are encoded by 44/105 ICEs (belonging to the Tn*916* superfamily) and 83/144 IMEs (belonging to class_1 to 4 and to class_7). Moreover, the diversity of TcpA CPs in IMEs is much larger than in ICEs (12 vs. 3 distinct families) which is reminiscent of relaxases (see above).

### High Diversity of Association of the Different Classes of Signature Genes within IMEs

Our analysis of the co-occurrence of relaxase superfamilies, CPs, and integrases in IMEs reveals some mandatory associations, consistent with the classification defined in section “New Relaxase Families Related to RCR Initiator Proteins in IMEs” (**Table 2**). Tyrosine integrases were found in all IMEs encoding non-canonical relaxases related to RCR initiators, i.e., (i) a Rel_PF02486/MobT relaxase or (ii) relaxases from the four new superfamilies identified in this study. On the other hand, serine integrases were found in all IMEs encoding relaxase from canonical Rel_PF03389/MobQ, Rel_PF13814/MobC, Rel_PF01076/MobV (in the absence of Rel_PF02486/MobT relaxase) and Rel_PF03432/MobP superfamilies. Furthermore, IMEs encoding a single Rel_PF02486/MobT or a relaxase belonging to one of the four new superfamilies also encode either TcpA or no CP. TcpA CPs were never found in IMEs containing one of the canonical relaxases (Rel_PF03389/MobQ, Rel_PF13814/MobC, Rel_PF01076/MobV (alone or with Rel_PF02486/MobT) and Rel_PF03432/MobP). These IMEs contain either no CPs (MobQ, MobV, and MobP) or CPs from the VirD4 superfamily (MobC).

**FIGURE 3:**
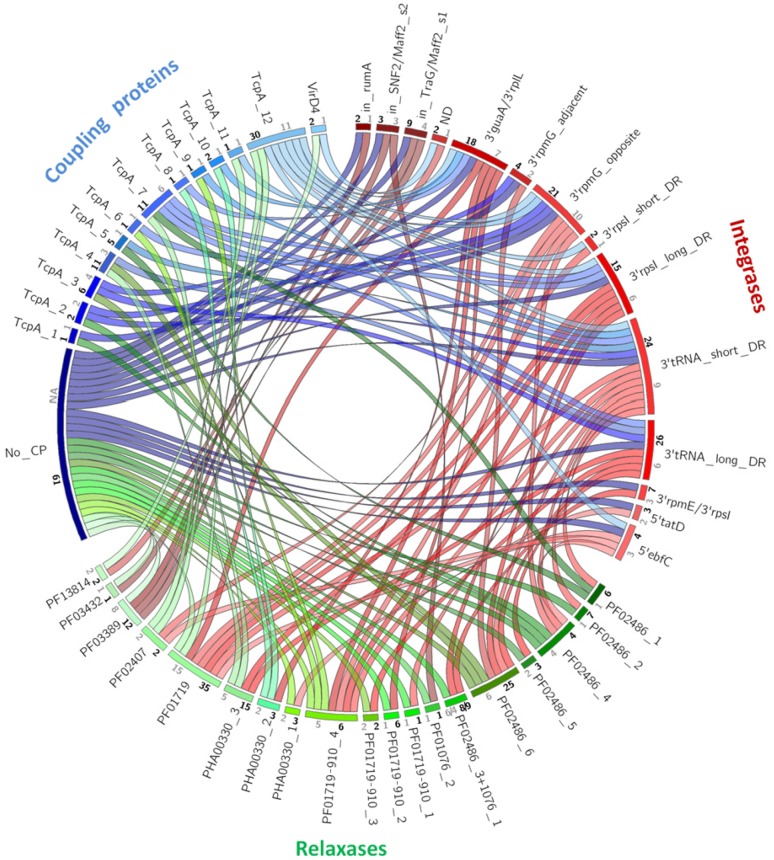
**Co-occurrence of relaxases, CPs and integrases among IMEs.** Arcs group proteins belonging to the same family according to phylogenetic analysis and 40% sequence identity clustering: red, green, and blue arcs for clustered integrases, relaxases, and CPs, respectively. Ribbons indicate the association between integrases and relaxases in red, relaxases and CPs in green and CPs and integrases in blue. Numbers in black show the number of IMEs belonging to the same family. Numbers in gray show the number of 90% sequence identity clusters within the family.

An illustration of all possible ternary Integrase–Relaxase–CP associations in our collection of 144 IMEs is shown in **Figure 3**. For each type of signature protein, the families identified by phylogenetic analyses and clustering at 40% identity are represented by arcs on the circle. The numbers of members in each family are reported in black. The numbers of clusters of sequences sharing more than 90% identity are reported in gray on each arc in order to estimate the sequence diversity in each family of signature protein. Apart from signature protein families with few representatives (*n* < 3) or with low diversity (90% identity clusters <3), this analysis of the co-occurrence of the different families of integrases, CPs, and relaxases (**Figure 3**) reveals no exclusive associations. Altogether, 39 different ternary associations were observed suggesting a high frequency of shuffling between signature proteins.

### Organization of Conserved CDSs in IMEs: Predominance of a Common Compact Structure

**FIGURE 4:**
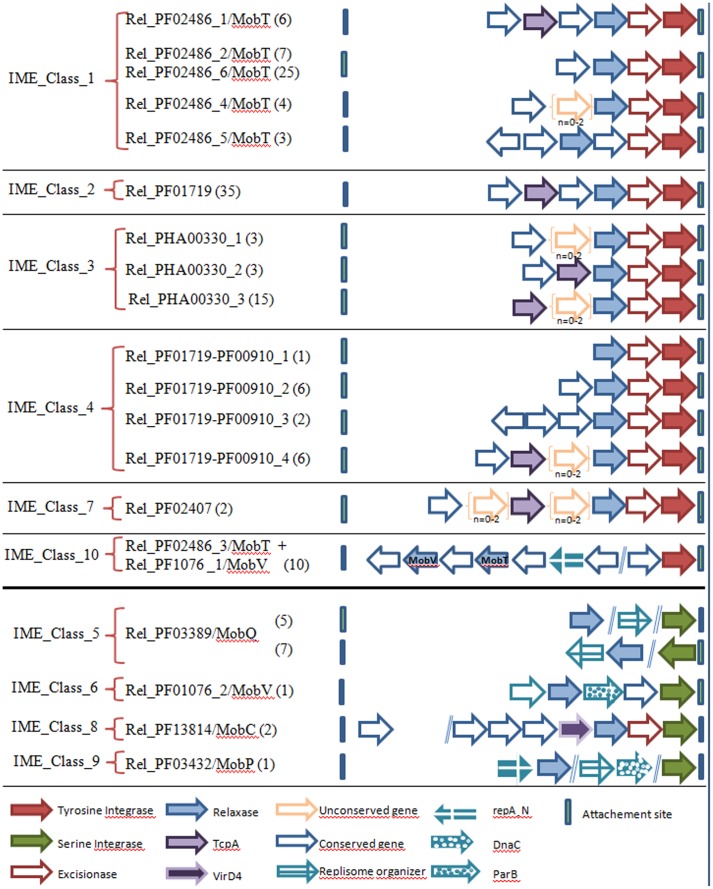
**Conserved CDS architectures within IMEs.** The classification of IMEs is based on their relaxase content (see also **Table 2**). The genes encoding conserved proteins in all IMEs sharing the same relaxase superfamily are indicated by arrows. The genes between brackets correspond to non-conserved genes located between conserved ones. The numerical values show the numbers of IMEs sharing the same structure.

For a better characterization of the IMEs, a search for conserved CDSs in IMEs encoding the same superfamily of relaxases was undertaken. The various conserved CDS architectures are schematized in **Figure 4**. Apart from IME_Class_10, the IMEs with a tyrosine integrase encode a unique non-canonical relaxase (Rel_PF02486/MobT, Rel_PF01719, Rel_PHA00330, Rel_PF01719-PF00910, and Rel_PF02407). These IMEs display a compact structure composed of successive genes (relaxase, excisionase, and integrase), generally preceded by a TcpA gene (83/118). Interestingly, the same compact structure was found in IME_Class_8, even if the encoded proteins are not related (a VirD4 CP instead of a TcpA CP, a canonical Rel_PF13814/MobC relaxase instead of relaxase related to RCR initiators and a serine integrase instead of tyrosine integrase).

All other classes of IMEs encode other types of canonical relaxases and do not encode a CP. In IME_Class_10, the two genes encoding relaxases (a canonical Rel_PF01076/MobV and a non-canonical Rel_PF02486/MobT) are located far upstream of the tyrosine integrase gene in the opposite orientation. As well as the two relaxases and a tyrosine integrase, these IMEs encode a protein with a repA_N domain that is also found in proteins that initiates the theta replication of plasmids and of ICEs belonging to the Tn*5252* superfamily from firmicutes (Weaver et al., 2009; Guerillot et al., 2013; Ambroset et al., 2016). However, repA_N proteins from IMEs are shorter (∼100 amino acids) than those found in plasmids and ICEs (∼340 amino acids), suggesting that they probably serve a different function.

Apart from IME_Class_8, all IMEs encoding a serine integrase and relaxases from canonical superfamilies (Rel_PF03389/MobQ, Rel_PF01076/MobV, and Rel_PF03432/MobP) also encode one or several proteins that could be involved in the maintenance of the excised elements. These proteins include homologs to: (i) ‘replisome organizers’ or ‘DnaC-related’ proteins involved in the initiation of theta replication of various phages from Firmicutes such as phi5218, phi4268, or phi9871 (Trotter et al., 2006; Tang et al., 2013; McDonnell et al., 2016), (ii) ‘ParB’ proteins involved in chromosome and plasmid partitioning (domain TIGR00180) (**Figure 4**). Such proteins are not encoded by any other class of IMEs analyzed in this study.

In summary, whereas the analysis of IME signature proteins shows a remarkable diversity, preventing their classification on the basis of their relationships, the analysis of their CDS organization shows that most of them (IME_Class_1, _2, _3, _4, _7, and _8, representing 120/144 IMEs) harbor a similar compact organization. This conserved compact organization was observed for all the IMEs encoding a single relaxase related to RCR initiators (Rel_PF02486/MobT type and four other new superfamilies discovered in this study) and for all IMEs encoding a CP (TcpA or VirD4).

### Modular Evolution of IMEs

**FIGURE 5:**
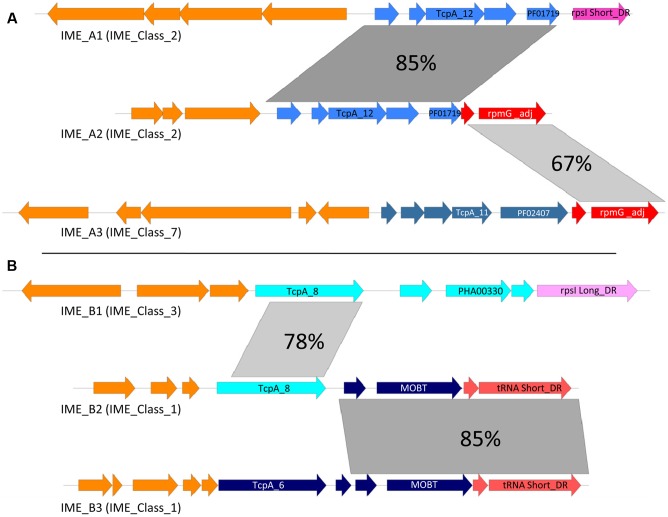
**Modular evolution of IMEs. (A)** Sequence comparison of IME_*A2* with IME_*A1* and IME_*A3*. **(B)** Sequence comparison of IME_*B2* with IME_*B1* and IME_*B3*. Percentages of nucleic identities are shown in gray. Blue: genes encoding conserved proteins of the mobilization module. Red or pink: genes encoding conserved proteins of integration/excision module. Orange: other genes. IME_*A1* = IME_*SdyRE378_rpsI*, IME_*A2* = IME_*SanC238_rpmG, IME_A3* = IME_*Sag201008_rpmG*, IME_*B1* = IME_*Spas43144_rpsI*, IME_*B2* = IME_*SanMAS624_tRNAlys and* IME_*B3* = IME_*SpnOXC141_rpmG.*

Sequence comparison of IMEs suggests that most of the exchanges of modules or CP-encoding genes occur between IMEs with similar structures, especially between elements harboring the compact structure described above. **Figure 5** illustrates two such situations involving IME_Class_2 and IME_Class_7 (**Figure 5A**), and IME_Class_3 and IME_Class1 (**Figure 5B**). In the first example, two members of IME_Class_2 (named here IME_*A1* and IME_*A2* for simplicity), exhibit a closely related mobilization module but their integration/excision modules are very different. Moreover, the integration module of IME_*A2* is related to that of IME_*A3* (from IME_Class_7) suggesting a probable exchange of integration modules between IMEs. In the second example, IME_*B2* and IME_*B3* (from IME_Class_1) have related integration and mobilization modules with the exception of their CP. However, the TcpA encoded by IME_*B2* is related to the one encoded by IME_*B1* (from IME_Class3). These data point to an exchange of genes encoding TcpA CPs between IMEs encoding different types of CPs and integrases.

## Discussion

### Prevalence and Diversity of IMEs

Integrative and mobilizable elements are by far the least known elements that transfer by conjugation. Indeed, until now, very little information on their prevalence has been available. Considering their diversity, very few IMEs with different mobilization and/or recombination modules have been reported (only 15 in 2013, see Bellanger et al., 2014 for a review). Here, we identified all IMEs carried by 124 genomes from 27 species of *Streptococcus* and compared their abundance and diversity to those of ICEs previously identified in the same set of strains (Ambroset et al., 2016). We demonstrated that IMEs have a very high prevalence and are about 40% more abundant than ICEs. We also found that the mobilization modules of IMEs display a larger diversity than the conjugation modules of ICEs.

Only 20% of our collection of streptococcal IMEs encode canonical relaxases (i.e., belong to the Rel_PF03389/MobQ, Rel_PF01076/MobV, Rel_PF13814/MobC, or Rel_PF03432/MobP superfamilies), already found to be encoded by conjugative and mobilizable elements from G- bacteria (Garcillan-Barcia et al., 2009). Among them, none encode any CP (IME_Class_5, _6, and _9) except the two IMEs with a Rel_PF13814/MobC relaxase (IME_Class_8) that encode a canonical VirD4 CP. This is reminiscent of mobilizable plasmids of G- bacteria and firmicutes (Garcillan-Barcia et al., 2009).

Unexpectedly, the large majority (90%, *n* = 129/144) of streptococcal IMEs in our collection encode non-canonical relaxases (Rel_PF02486/MobT, Rel_PF01719, Rel_PHA00330, Rel_PF01719-PF00910, and Rel_PF02407) related to proteins responsible for RCR initiation involved in the maintenance of plasmids from firmicutes or viruses. MobT was previously identified in a few predicted IMEs (Bellanger et al., 2014) and in ICEs belonging to the Tn*916* superfamily (Ambroset et al., 2016). The four other superfamilies of putative relaxases are unrelated to any known relaxase of mobilizable or conjugative elements. It should be emphasized that the MobT “relaxase” of the integrative and conjugative element ICE*Bs1* from the firmicute *Bacillus subtilis* is involved in not only the initiation of the conjugative transfer of ICE*Bs1* but also the initiation of RCR needed for the maintenance of ICE*Bs1* after excision (Lee et al., 2010). In the same way, another family of “RCR initiators” exhibiting a Rep_1/PF01446 domain is involved not only in the maintenance of three plasmids from firmicutes but also in their mobilization by ICE*Bs1* (Lee et al., 2012). Therefore, the classical distinction between RCR initiators and relaxases could lose its relevance. In IMEs encoding a CP (*n* = 85/144), RCR initiator-related relaxases are always associated with a non-canonical TcpA CP. The strict association of RCR initiator and TcpA is also observed in conjugative elements encoding a RCR relaxase (i.e., the ICEs belonging to the Tn*916/*ICE*Bs1*/ICE*St3* from firmicutes) (Guglielmini et al., 2013; Ambroset et al., 2016). In these IMEs and ICEs, the *tcpA* gene is located upstream from the RCR “relaxase” gene. Taken together, these findings reveal that non-canonical relaxases related to four types of RCR initiators and non-canonical CPs related to FtsK DNA translocase are involved in the mobilization of most streptococcal IMEs.

FtsK-related proteins were previously found to be encoded by various small plasmids mainly from firmicutes (see for examples, NCBI reference sequences NP_203541, NP_613077, YP_251910, WP_011669127, YP_001967631, YP_006939188) but they were never proposed to be mobilization CPs (see for example Bachrach et al., 2004; Bjorland et al., 2007; Shkoporov et al., 2008). However, according to our analysis using CONJscan-T4SSscan, all these proteins belong to the TcpA family. Interestingly, none of these plasmids encode a canonical relaxase. Rather, they carry a “RCR initiator” gene (located next to the *tcpA*) encoding either one of the domains found in IME relaxases or a PF01446 domain (i.e., the domain found in RCR initiator/relaxases from the small plasmids mobilized by ICE*Bs1*). Therefore, it seems highly probable that these small plasmids are mobilizable. As previously proposed by Lee et al. (2012), it is probable that at least some of the small plasmids from firmicutes encoding a “RCR initiator” and lacking any canonical relaxase gene, CP, or T4SS protein, could be also mobilizable. Finally, it should also be noted that many plasmids devoid of relaxase could also carry an *oriT* related to those of conjugative element and therefore could be mobilizable *in trans*, as recently found for most plasmids from staphylococci (O’Brien et al., 2015; Pollet et al., 2016). Taken together, all these data point to a previous underestimation of the number of mobilizable plasmids and instead support their very high prevalence.

Besides IMEs and ICEs, our analysis of streptococcal genomes reveals many elements that (i) are flanked by DRs and (ii) encode an integrase but (iii) are devoid of CP and of canonical or RCR initiator-related relaxase (data not shown). At least some of these could correspond to IMEs. This hypothesis is supported first by the discovery of a novel type of relaxase related to tyrosine recombinases that is encoded by the pCW3 conjugative plasmid from the firmicute *C. perfringens* (Wisniewski et al., 2016). Second, in proteobacteria, IMEs devoid of relaxase but carrying an *oriT* are found to be mobilizable (Daccord et al., 2010) and one such IME (IME MTn*Sag1*) have previously been described in *S. agalactiae* (Achard and Leclercq, 2007). Thus, the prevalence and diversity of IMEs within analyzed streptococcal genomes could be even greater than that described here.

### Modular and Intramodular Evolution

The comparison of phylogenetic analyses of integrases with those of relaxases and CPs reveals many inconsistencies, probably due to multiple replacements of integration/excision or mobilization modules between IMEs. Such replacements were previously observed in streptococcal ICEs (Ambroset et al., 2016). The phylogenetic analysis of integrase families (**Figure 1** and **Supplementary Figure S1**) clearly shows that replacement of integration/excision modules can occur not only between IMEs or between ICEs but also between ICEs and IMEs.

Within the mobilization modules encoding non-canonical relaxases, the comparison of phylogenetic analyses and/or co-occurrence of relaxases and TcpA CPs reveals inconsistencies. Thus, 10 out of 25 IMEs encoding a MobT relaxase that belong the PF02486_6 family do not carry a *tcpA* gene or pseudogene, whereas the 15 others have one. The distribution of these latter suggests that an ancestral IME devoid of TcpA has recently acquired a *tcpA* gene. On the contrary, among IMEs encoding relaxases belonging to the PF01719, PF1719-PF00910, and PHA00330 superfamilies, only 13 do not encode a TcpA and probably lost their *tcpA* by deletion. In some cases, almost identical IME relaxases (see for example the Rel_PF02486_6 cluster in **Supplementary Figure S2**) are associated with TcpA from different families suggesting that intramodular gene replacements occurred. In most cases, the data does not allow us to determine precisely what happened. However, the phylogenetic tree of the PF02486_6 relaxases suggests a replacement of a TcpA_5 CP by a TcpA_6 CP. Interestingly, although two superfamilies of relaxase (Rel_PF02486/MobT and Rel_PF03432/MobP) and the two superfamilies of CPs (VirD4 and TcpA) are shared by ICEs and IMEs from streptococci, there is no evidence of exchange of these genes between ICEs and IMEs.

Various IMEs integrated in *rpsI*, *rpmG*, and *rplL* were found to be integrated in tandem with other IMEs or ICEs encoding either related, distantly related, or unrelated integrases, relaxases, and/or CPs. We have also found many decayed elements or genomic islands in accretion with streptococcal IMEs (data not shown). These accretions result from the integration of an incoming IME or ICE by site-specific recombination in the *attL* or *attR* site of related or unrelated resident element that may not be followed by a deletion (Pavlovic et al., 2004; Bellanger et al., 2011). An accretion between elements targeting the same insertion gene and subsequent deletion of one of the transfer modules and one of the recombination modules is probably responsible for a large part of the replacement of modules or TcpA genes.

### Are TcpA CP Needed or not for Mobilization?

In fact, the non-canonical TcpA superfamily of CPs was previously reported to be encoded by conjugation modules from firmicutes, but TcpA has not yet been found in a mobilization module. In conjugative elements, TcpA proteins are associated with non-canonical relaxases: (i) the relaxase of the conjugative pCW3 plasmid from *C. perfringens* that is related to tyrosine recombinases (Wisniewski et al., 2016) or (ii) the MobT relaxases from the ICEs belonging to ICE*Bs1*/Tn*916*/ICE*St3* superfamily that are related to RCR initiators. In this work, we identified many IMEs encoding a TcpA CP: all of them encode a non-canonical relaxase. Although all IMEs encoding a non-canonical relaxase have a similar organization, their mobilization modules are highly versatile. First, closely related relaxases can be associated or not with a TcpA CP, suggesting that IME-encoded CP might not be needed for mobilization. If so, it can be hypothesized that the non-canonical relaxase might interact with the T4SS of the mobilizing conjugative element, either *via* the CP encoded by the conjugative element or *via* its cognate CP. In this hypothesis, the IME-encoded TcpA might enhance the mobilization efficiency and/or enlarge the mobilization range. Second, closely related relaxases can be associated with different distantly related TcpA CPs, indicating that the IME relaxase can interact with distantly related CPs. We hypothesize that the change of CP might have an impact on the mobilization efficiency and/or range.

### IMEs within ICEs, a New Mobilization Mechanism?

The IMEs from streptococci carry diverse recombination modules and have a large array of integration specificity. Almost all serine integrases from IMEs catalyze site-specific integration within genes leading to their disruption. Interestingly, the majority of IME serine integrases (12/16) specifically target several conserved genes from the Tn*5252* superfamily, a group of ICEs that is widespread in streptococci. As previously discussed for ICEs (Ambroset et al., 2016), we would expect that target specificity should be selected to have the least effect on host fitness. Here, the disruption of a conjugation gene would have little or no effect on bacterial host but would be deleterious or lethal for the host ICE. For instance, insertion in *traG*, that encodes a VirD4 CP, would abolish the ICE transfer and therefore the mobilization of the IME by the host ICE. The consequences of the integration/excision balance of ICE or IME encoding serine recombinases have never been studied but are documented for some prophages encoding serine recombinase. For these prophages, excision occurs not only during the activation of lytic phase but also when expression of the host target gene is needed (Kunkel et al., 1990; Rabinovich et al., 2012). By analogy, we can hypothesize that excision of IMEs integrated within specific conjugation genes of ICEs would be caused by the induction of conjugation. After IME excision, the conjugation module would be functional and could be expressed, thus allowing ICE transfer. The IME could use the CP and T4SS of this ICE to transfer and then could integrate in the ICE in the transconjugant. Furthermore, if the ICE that primarily hosts the IME does not transfer or integrate in the recipient cell, the incoming IME could integrate in another resident element (related ICE or decayed ICE as long as it carries the IME integration site). This would explain the presence of such IMEs in many decayed ICEs from streptococci. ICE*Sp2905* from *S. pyogenes*, an ICE integrated in *rumA*, was demonstrated to transfer (Giovanetti et al., 2012) although it carries two IMEs: one integrated in *snf2* and the other in *maff2* (Bellanger et al., 2014). Although we cannot exclude that the disrupted genes are not required for ICE transfer, we can also hypothesize that the transfer could be divided into successive stages including excisions of the ICE and IMEs, independent transfers of the IMEs and of the ICE devoid of IMEs, insertions of the transferred ICE in *rumA* site and insertion of the IMEs within the transferred ICE. Such a mobilization mechanism of an IME integrated in an ICE can also be proposed for IMEs encoding tyrosine integrases that are site-specifically integrated in the putative *oriT* from ICEs belonging to Tn*916* and ICE*St3* families that were found in *S. agalactiae* and in *S. mutans* (Puymege et al., 2015).

### Replication of Excised IMEs

Integrative and mobilizable elements and ICEs are integrated in the chromosome and are transmitted to the daughter cell as part of the chromosome. However, an excised IME would be lost in one of the daughter cell during cell division in the absence of replication. Several recent studies indicated that extrachromosomal replication is involved in the maintenance of various ICEs transferring as single-strand DNA (Ramsay et al., 2006; Lee et al., 2012; Guerillot et al., 2013; Carraro et al., 2015), but data suggesting replication of excised IMEs has not been reported so far. All streptococcal IMEs encoding a Rel_PF03432/MobP relaxase and most of those encoding a Rel_PF03389/MobQ carry one or two genes encoding proteins downstream from the relaxase gene that are distantly related to primosome proteins. Such proteins are known to be responsible for the initiation of the theta replication of prophages. Altogether, these data suggest that these IMEs are able to replicate with a theta mechanism after excision. Ten other IMEs (IME_Class_10 family) sharing a similar organization encode two putative relaxases. One belongs to Rel_PF02486/MobT superfamily, i.e., a family including both RCR initiators and relaxases. The other belongs to Rel_PF01076/MobV family. The structure of the region carrying these genes is reminiscent of the one of numerous mobilizable plasmids from firmicutes where the Rel_PF01076/MobV protein was found to be involved in mobilization whereas the Rel_PF02486/MobT protein is involved in RCR (Lorenzo-Diaz et al., 2014). Accordingly, the Rel_PF01076/MobV protein of an IME of the IME_Class_10 family was very recently shown to be a mobilization relaxase (Lorenzo-Diaz et al., 2016). Therefore, it is likely that the Rel_PF01076/MobV protein is the IME relaxase, whereas the Rel_PF02486/MobT protein would be involved in RCR replication of the excised IME. Alternatively, as previously found for the MobT relaxase of ICE*Bs1* (Lee et al., 2012), the Rel_PF02486/MobT protein could ensure both functions. Finally, all IMEs encoding a single Rel_PF01076/MobV relaxase encode a protein related to the ParB proteins that could be involved in the partition of the excised IME. Taken together, these data suggest that almost all streptococcal IMEs encoding canonical relaxases (except the two elements encoding a MobC relaxase) encode proteins involved in the maintenance of their excised forms. Such proteins were not found for any IME encoding single non-canonical relaxases such as MobT. Since the MobT protein from ICE*Bs1* is both the relaxase and the initiator of RCR involved in the maintenance of excised ICE*Bs1* (Lee et al., 2012), we cannot exclude the possibility that the five superfamilies of non-canonical relaxases might have both functions. Overall, many if not all IMEs and ICEs might exist in two states, namely a main dormant integrated state and an activated excised state which would be maintained by replication.

## Author Contributions

GG and SP conceived the reference database of signature proteins. CC, NL-B, GG, and M-DD contributed to the conception of the work. CC, GG, NL-B, CA, M-DD, VL, and TL performed the acquisition and analysis of the data. GG, NL-B, CC, and M-DD drafted the manuscript. CC, NL-B, GG, and SP elaborated the figures, tables, and references. All authors criticized and finally approved the final version of the manuscript.

## Conflict of Interest Statement

The authors declare that the research was conducted in the absence of any commercial or financial relationships that could be construed as a potential conflict of interest.

## Footnotes

1 http://www.ncbi.nlm.nih.gov/genome/browse/, last accessed December 2013

2 http://central.biomart.org/

3 http://www.ncbi.nlm.nih.gov/Structure/cdd/wrpsb.cgi

4 http://mobyle.pasteur.fr/cgi-bin/portal.py\#forms::CONJscan-T4SSscan

5 https://toolkit.tuebingen.mpg.de/blastclust

6 http://circos.ca/

## Supplementary Material

FIGURE S1 **Phylogenetic tree of serine integrases.** One representative of each 90% protein identity cluster from IMEs (in black) and one representative of each 90% protein identity cluster of serine integrases from ICEs targeting the same site as IMEs (in mauve and underlined) are presented in the ML tree. Bootstrap values (BioNJ/ML) are given only when they exceed 50 for both analyses. The target gene is mentioned in the IME/ICE names. Serine integrases sharing more than 40% identity and therefore belonging to the same family are merged with brackets. These families are distinguished with different numbers. Refer to Supplementary Table S1 for IME and strain details.

FIGURE S2 **Phylogenetic tree of Rel_PF02486/MobT relaxases.** All the Rel_PF02486/MobT relaxases from IMEs (in black) and only one representative of each 90% protein identity cluster of MobT relaxases from ICEs (in mauve and underlined) are presented in the ML tree. Bootstrap values (BioNJ/ML) are given only when they exceed 50 for both analyses. Relaxases sharing more than 40% sequence identity and therefore belonging to the same family are merged with brackets. These families are distinguished with a number preceded by the Pfam identifier of the characteristic domain of this superfamily. The TcpA family associated with each relaxase is indicated.

FIGURE S3 **Phylogenetic tree of Rel_PF01719 relaxases.** All the Rel_PF01719 relaxases are presented in the ML tree. Bootstrap values (BioNJ/ML) are given only when they exceed 50 for both analyses. All these relaxases share more than 40% sequence identity and therefore belong to a unique family. The TcpA family associated with each relaxase is indicated.

FIGURE S4 **Phylogenetic tree of Rel_PHA00330 relaxases.** All the Rel_PHA00330 relaxases are presented in the ML tree. Bootstrap values (BioNJ/ML) are given only when they exceed 50 for both analyses. The relaxases sharing more than 40% sequence identity and therefore belonging to the same family are merged with brackets. These families are distinguished with a number preceded by the identifier of the characteristic domain of this superfamily. The TcpA family associated with each relaxase is indicated.

FIGURE S5 **Phylogenetic tree of Rel_PF01719-PF00910 relaxases.** All the Rel_PF01719-PF00910 relaxases are presented in the ML tree. Bootstrap values (BioNJ/ML) are given only when they exceed 50 for both analyses. The relaxases sharing more than 40% sequence identity and therefore belonging to the same family are merged with brackets. These families are distinguished with a number preceded by the pfam identifier of the characteristic domains of this superfamily. The TcpA family associated with each relaxase is indicated.

FIGURE S6 **Phylogenetic tree of TcpA proteins.** All the TcpA CPs from IMEs (in black) and one of each 90% protein identity cluster of TcpA from ICEs (in mauve and underlined) are presented in the BioNJ tree. Bootstrap values are given only when they exceed 50. The TcpA CPs sharing more than 40% sequence identity and therefore belonging to the same family are merged with brackets. These families are distinguished with different numbers. The relaxases families associated with each TcpA family are indicated.
