# Supplementary figures and images for: A Glimpse into the World of Integrative and Mobilizable Elements in Streptococci Reveals an Unexpected Diversity and Novel Families of Mobilization Proteins
Source: Front Microbiol. 2017 Mar 20;8:443. doi: 10.3389/fmicb.2017.00443 (PMC5357655; doi:10.3389/fmicb.2017.00443)

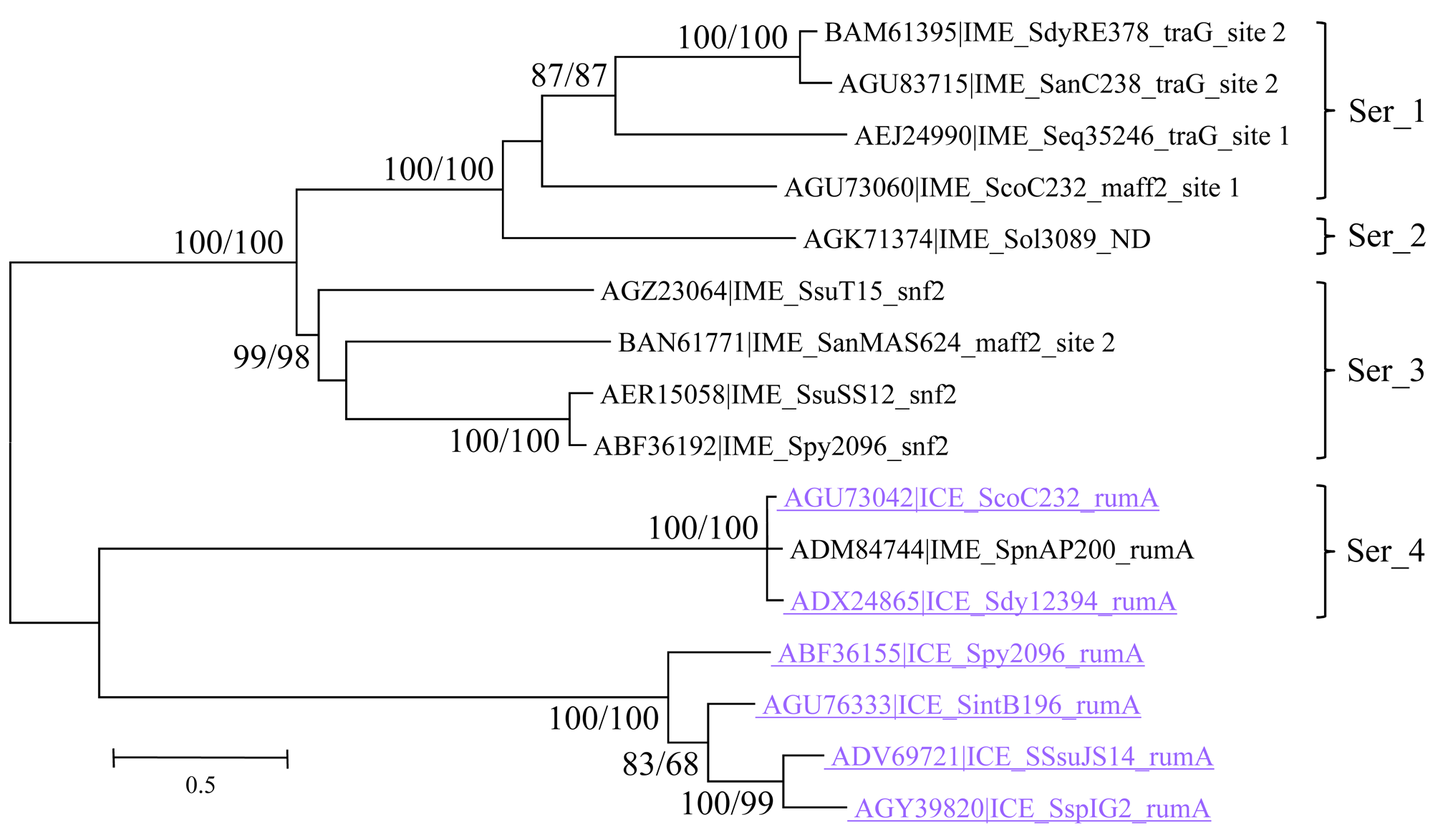

Supplement: FIGURE S1 — Phylogenetic tree of serine integrases. One representative of each 90% protein identity cluster from IMEs (in black) and one representative of each 90% protein identity cluster of serine integrases from ICEs targeting the same site as IMEs (in mauve and underlined) are presented in the ML tree. Bootstrap values (BioNJ/ML) are given only when they exceed 50 for both analyses. The target gene is mentioned in the IME/ICE names. Serine integrases sharing more than 40% identity and therefore belonging to the same family are merged with brackets. These families are distinguished with different numbers. Refer to Supplementary Table S1 for IME and strain details. [file Image_1.TIF]

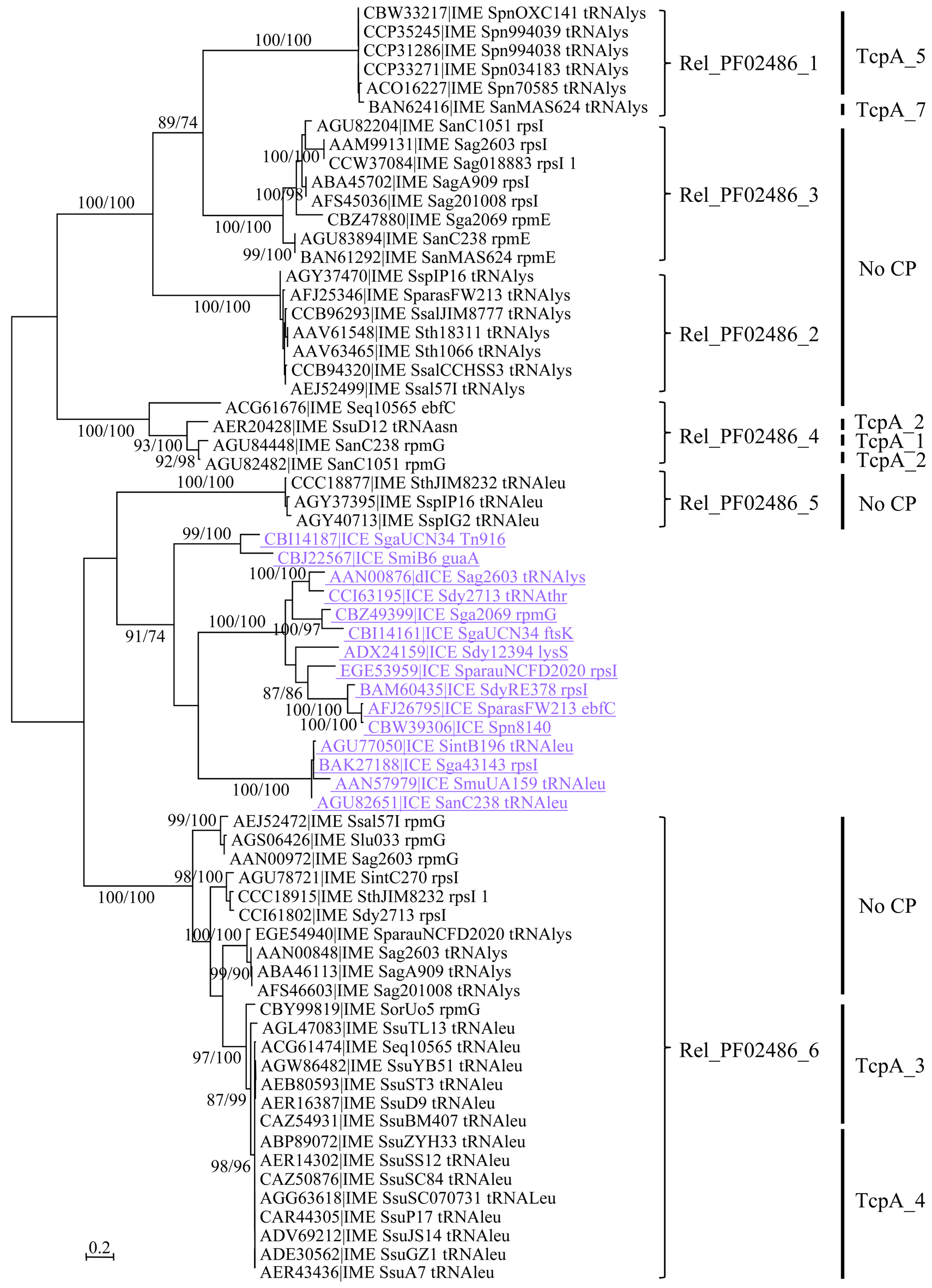

Supplement: FIGURE S2 — Phylogenetic tree of Rel_PF02486/MobT relaxases. All the Rel_PF02486/MobT relaxases from IMEs (in black) and only one representative of each 90% protein identity cluster of MobT relaxases from ICEs (in mauve and underlined) are presented in the ML tree. Bootstrap values (BioNJ/ML) are given only when they exceed 50 for both analyses. Relaxases sharing more than 40% sequence identity and therefore belonging to the same family are merged with brackets. These families are distinguished with a number preceded by the Pfam identifier of the characteristic domain of this superfamily. The TcpA family associated with each relaxase is indicated. [file Image_2.TIF]

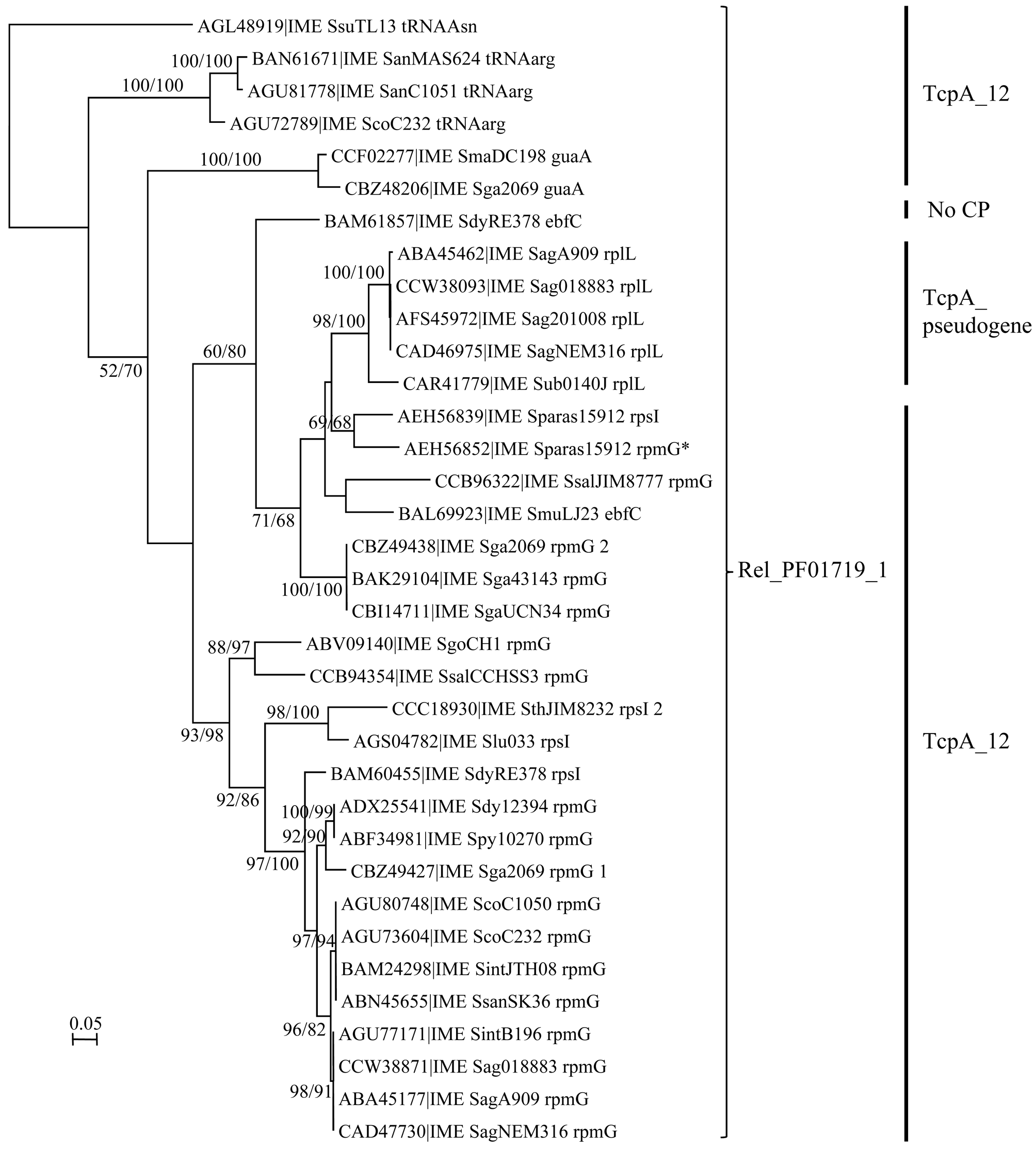

Supplement: FIGURE S3 — Phylogenetic tree of Rel_PF01719 relaxases. All the Rel_PF01719 relaxases are presented in the ML tree. Bootstrap values (BioNJ/ML) are given only when they exceed 50 for both analyses. All these relaxases share more than 40% sequence identity and therefore belong to a unique family. The TcpA family associated with each relaxase is indicated. [file Image_3.TIF]

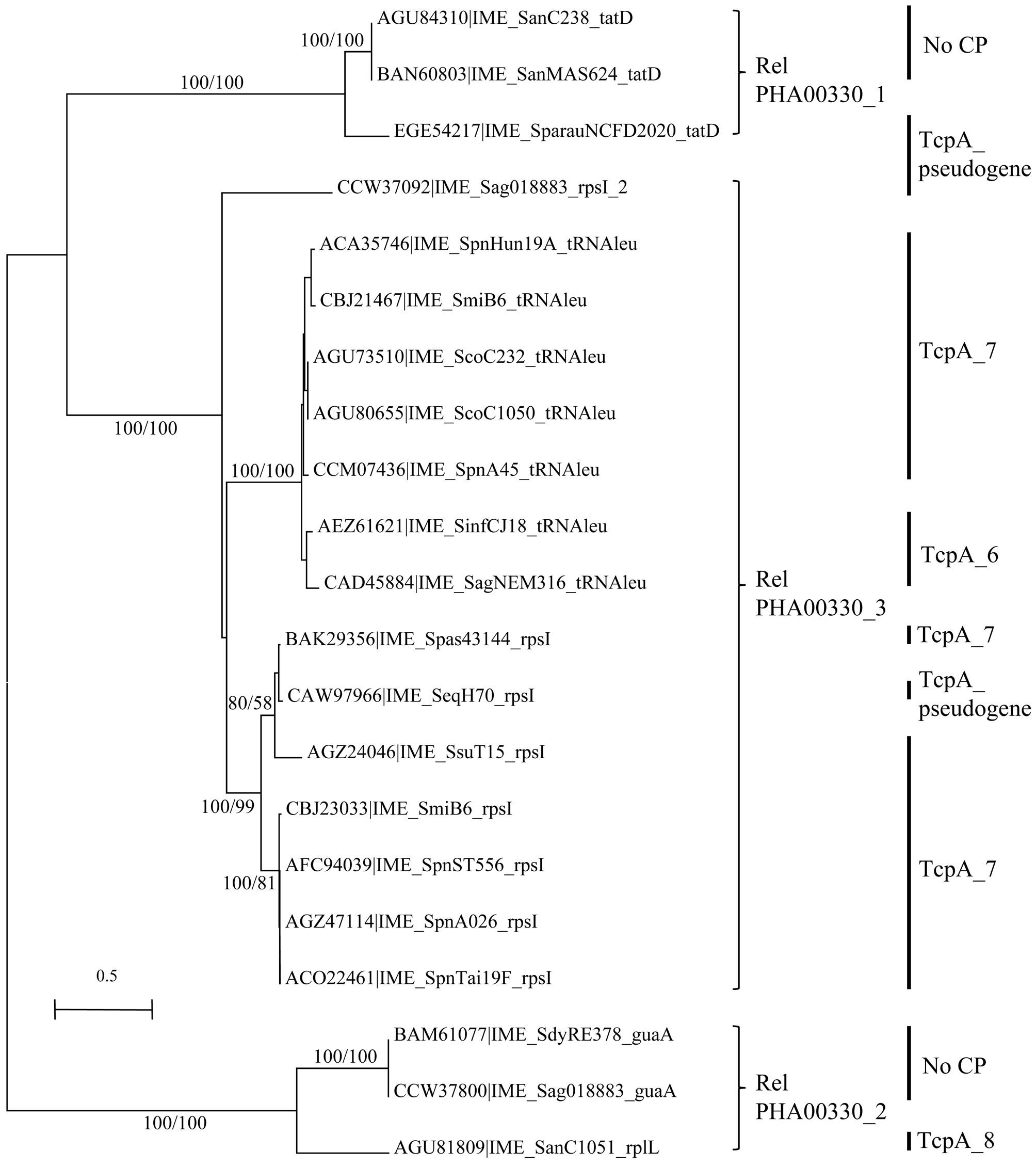

Supplement: FIGURE S4 — Phylogenetic tree of Rel_PHA00330 relaxases. All the Rel_PHA00330 relaxases are presented in the ML tree. Bootstrap values (BioNJ/ML) are given only when they exceed 50 for both analyses. The relaxases sharing more than 40% sequence identity and therefore belonging to the same family are merged with brackets. These families are distinguished with a number preceded by the identifier of the characteristic domain of this superfamily. The TcpA family associated with each relaxase is indicated. [file Image_4.TIF]

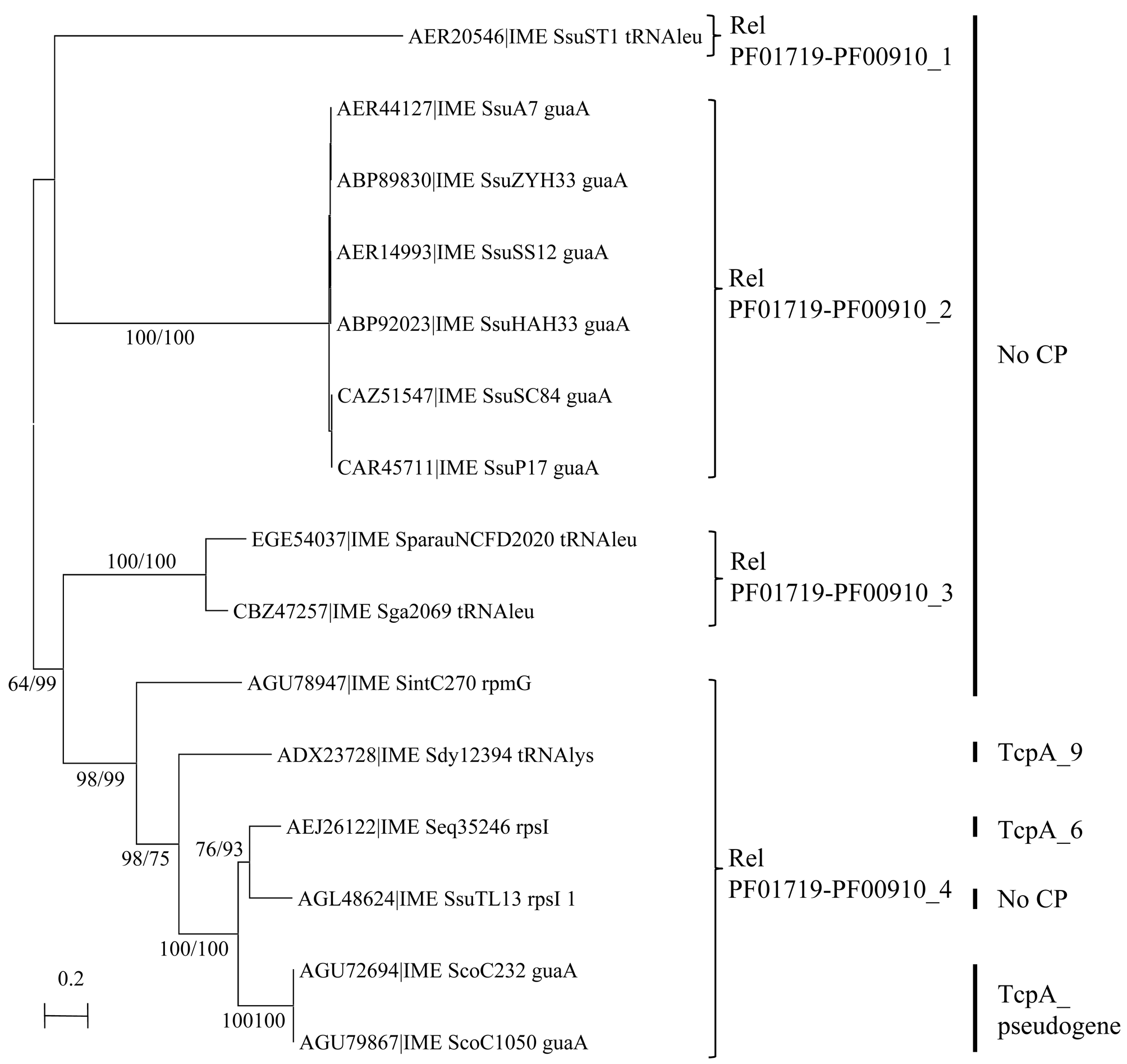

Supplement: FIGURE S5 — Phylogenetic tree of Rel_PF01719-PF00910 relaxases. All the Rel_PF01719-PF00910 relaxases are presented in the ML tree. Bootstrap values (BioNJ/ML) are given only when they exceed 50 for both analyses. The relaxases sharing more than 40% sequence identity and therefore belonging to the same family are merged with brackets. These families are distinguished with a number preceded by the pfam identifier of the characteristic domains of this superfamily. The TcpA family associated with each relaxase is indicated. [file Image_5.TIF]

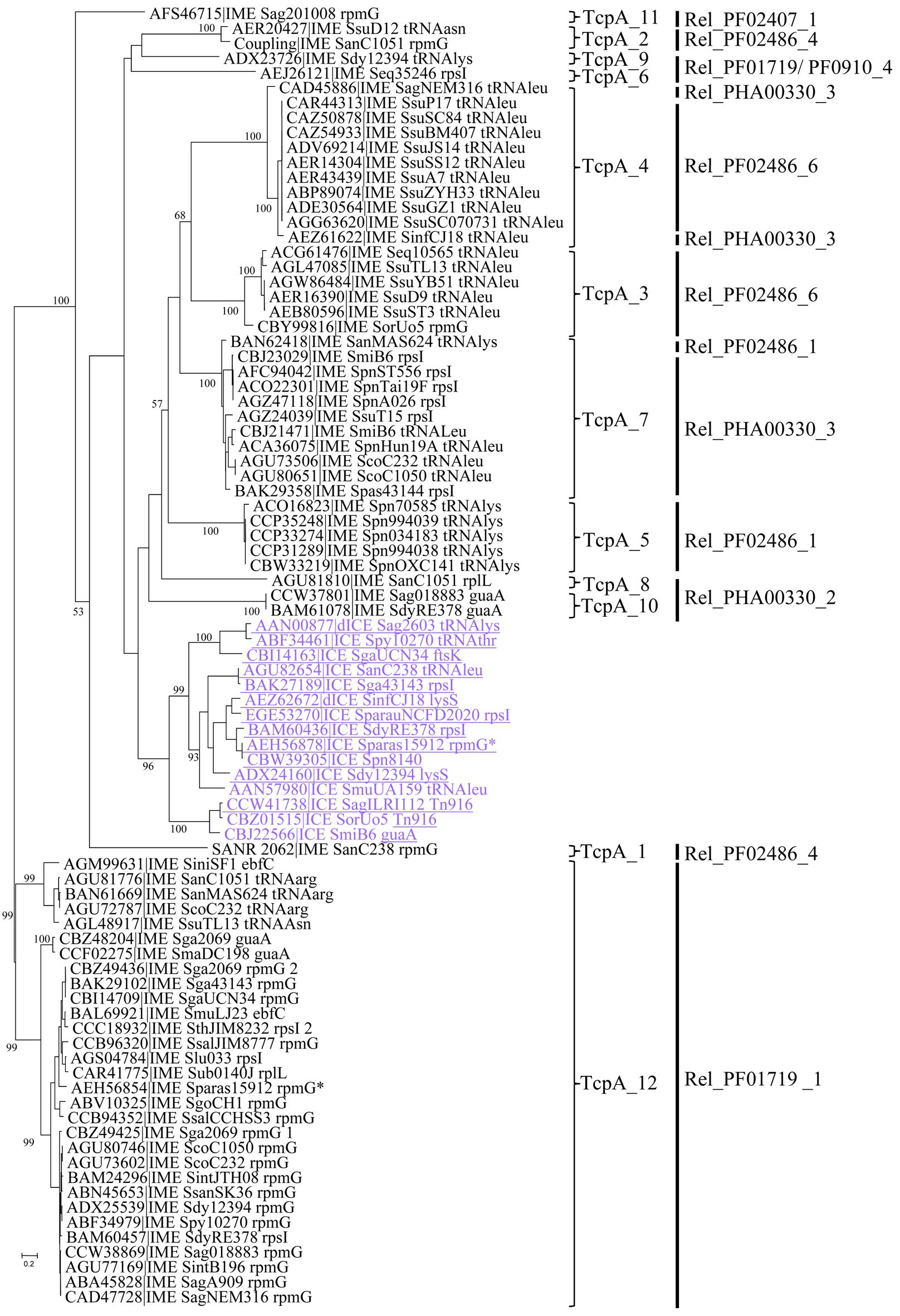

Supplement: FIGURE S6 — Phylogenetic tree of TcpA proteins. All the TcpA CPs from IMEs (in black) and one of each 90% protein identity cluster of TcpA from ICEs (in mauve and underlined) are presented in the BioNJ tree. Bootstrap values are given only when they exceed 50. The TcpA CPs sharing more than 40% sequence identity and therefore belonging to the same family are merged with brackets. These families are distinguished with different numbers. The relaxases families associated with each TcpA family are indicated. [file Image_6.TIF]
